# *N*-(2-Hy­droxy­benz­yl)adamantan-1-aminium 4-methyl­benzene­sulfonate

**DOI:** 10.1107/S1600536811048860

**Published:** 2011-11-19

**Authors:** Shao-Gang Hou

**Affiliations:** a Department of Chemical & Environmental Engineering, Anyang Institute of Technology, Anyang 455000, People’s Republic of China

## Abstract

In the crystal structure of the title salt, C_17_H_24_NO^+^·C_7_H_7_O_3_S^−^, the *N*-(2-hy­droxy­benz­yl)adamantan-1-aminium cations and 4-methyl­benzene­sulfonate anions are linked by O—H⋯O and N—H⋯O hydrogen bonds. C—H⋯π inter­actions are also observed between the cation and the anion.

## Related literature

For related compounds, see: Blagden *et al.* (2008 ▶); Vishweshwar *et al.* (2006 ▶); Kapildev *et al.* (2011 ▶); Schultheiss & Newman (2009 ▶).
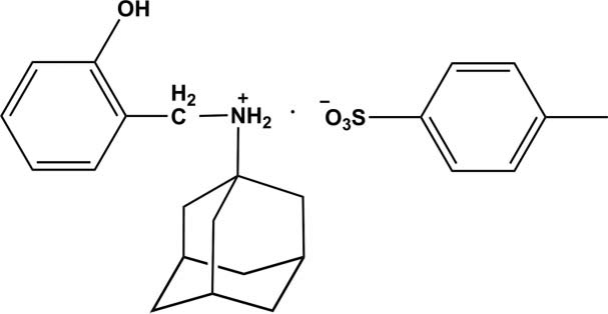


## Experimental

### 

#### Crystal data

C_17_H_24_NO^+^·C_7_H_7_O_3_S^−^
*M* *_r_* = 429.57
Triclinic, 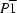

*a* = 10.159 (2) Å
*b* = 10.413 (2) Å
*c* = 11.270 (2) Å
α = 79.03 (3)°
β = 69.06 (3)°
γ = 78.49 (3)°
*V* = 1081.8 (4) Å^3^
*Z* = 2
Mo *K*α radiation
μ = 0.18 mm^−1^
*T* = 298 K
0.30 × 0.25 × 0.15 mm

#### Data collection

Rigaku Mercury2 diffractometer
Absorption correction: multi-scan (*CrystalClear*; Rigaku, 2005 ▶) *T*_min_ = 0.90, *T*_max_ = 0.99
11276 measured reflections
4940 independent reflections
3641 reflections with *I* > 2σ(*I*)
*R*_int_ = 0.051

#### Refinement

*R*[*F* ^2^ > 2σ(*F* ^2^)] = 0.055
*wR*(*F* ^2^) = 0.157
*S* = 1.08
4940 reflections
299 parameters
H-atom parameters constrained
Δρ_max_ = 0.30 e Å^−3^
Δρ_min_ = −0.37 e Å^−3^

### 

Data collection: *CrystalClear* (Rigaku, 2005 ▶); cell refinement: *CrystalClear*; data reduction: *CrystalClear*; program(s) used to solve structure: *SHELXTL* (Sheldrick, 2008 ▶); program(s) used to refine structure: *SHELXTL*; molecular graphics: *SHELXTL*; software used to prepare material for publication: *SHELXTL*.

## Supplementary Material

Crystal structure: contains datablock(s) I, global. DOI: 10.1107/S1600536811048860/xu5389sup1.cif

Structure factors: contains datablock(s) I. DOI: 10.1107/S1600536811048860/xu5389Isup2.hkl

Supplementary material file. DOI: 10.1107/S1600536811048860/xu5389Isup3.cml

Additional supplementary materials:  crystallographic information; 3D view; checkCIF report

## Figures and Tables

**Table 1:** Hydrogen-bond geometry (Å, °) *Cg* is the centroid of the C18–C23 benzene ring.

*D*—H⋯*A*	*D*—H	H⋯*A*	*D*⋯*A*	*D*—H⋯*A*
N1—H1*A*⋯O2^i^	0.90	2.09	2.922 (2)	154
N1—H1*B*⋯O3	0.90	2.05	2.860 (2)	150
O4—H4*A*⋯O2^ii^	0.82	1.90	2.719 (2)	180
C14—H14*A*⋯*Cg*	0.97	2.75	3.668 (3)	158

Symmetry codes: (i) 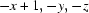; (ii) 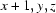.

**Fig. 1.**
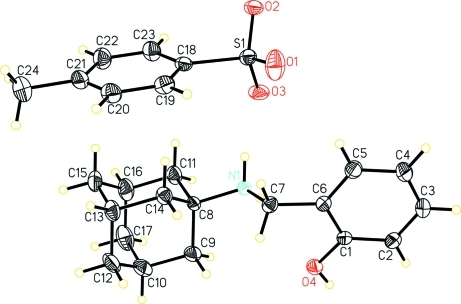
Molecular view of the title compound with the atomic numbering scheme. Displacement ellipsoids are drawn at the 30% probability level.

**Fig. 2.**
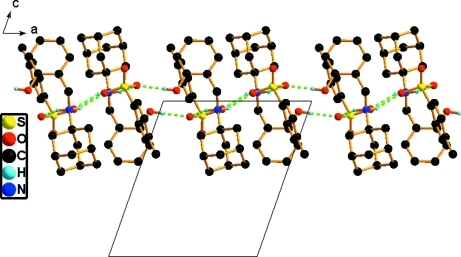
The crystal packing of the title compound viewed along the b axis showing the one-dimensionnal hydrogen bondings chain (dashed line).

## supplementary crystallographic information

### Comment

Organic salts are becoming increasingly important as new molecule-based crystalline materials with the potential to provide optimal physical properties whilst retaining the chemical properties of the organic components (Blagden *et al.*, 2008; Vishweshwar *et al.*, 2006). Physicochemical properties such as the melting point, stability and solubility can be tuned through crystallization (Kapildev *et al.*, 2011; Schultheiss & Newman, 2009). The synthesis of organic salts often relies on the acid-amide H-bonds interactions. Herein, we report the crystal structure of the title compound, N-(2-hydroxybenzyl)adamantan-1-aminium 4-methylbenzenesulfonate.

The asymmetric unit is composed of one N-(2-Hydroxybenzyl)adamantan-1-aminium cation and one 4-methylbenzenesulfonate anion. The amine N1 atom was protonated. And the sulfonic group was deprotonated to keep the charge balance. The two benzene rings are nearly perpendicular and twisted from each other by a dihedral of 83.11 (1)°. The geometric parameters of the title compound are in the normal range.

In the crystal structure, all the hydroxy and amino H atoms are involved in intermolecular O—H···O and N—H···O hydrogen bonds interactions with the sulfonic O atoms. These hydrogen bonds link the molecules into an one-dimentional chain parallel to the *a*-axis (Table 1 and Fig.2).

### Experimental

A mixture of N-(2-hydroxybenzyl)adamantan-1-amine (2.0 mmol), 4-methylbenzenesulfonic acid (2.0 mL) and 20 mL distilled water were added into a 50 ml flask and refluxed for 5 h, then cooled and filtrated. The solution was evaporated slowly in the air. Colorless block crystals suitable for X-ray analysis were obtained after one week.

### Refinement

All H atoms attached to C atoms were fixed geometrically and treated as riding with C–H = 0.93 Å (aromatic), C–H = 0.97 Å (methylene), C–H = 0.98 Å (methine) and C–H = 0.96 Å (methyl) with *U*_iso_(H) = 1.2*U*_eq_(C) and *U*_iso_(H) = 1.5*U*_eq_(methyl). H atoms bonded to N and O atoms were located in a difference Fourier map and restrained with the H—N1 = 0.90 (2)Å and H—O4 = 0.82 (2)Å. In the last stage of refinement they were treated as riding on the N and O atoms with *U*_iso_(H) = 1.5*U*_eq_(N,O).

### Crystal data

**Table:**

C_17_H_24_NO^+^·C_7_H_7_O_3_S^−^	*Z* = 2
*M**_r_* = 429.57	*F*(000) = 460
Triclinic, *P*1	*D*_x_ = 1.319 Mg m^−^^3^
Hall symbol: -P 1	Mo *K*α radiation, λ = 0.71073 Å
*a* = 10.159 (2) Å	Cell parameters from 4940 reflections
*b* = 10.413 (2) Å	θ = 3.2–27.5°
*c* = 11.270 (2) Å	µ = 0.18 mm^−^^1^
α = 79.03 (3)°	*T* = 298 K
β = 69.06 (3)°	Block, colorless
γ = 78.49 (3)°	0.30 × 0.25 × 0.15 mm
*V* = 1081.8 (4) Å^3^	

### Data collection

**Table:**

Rigaku Mercury2 diffractometer	4940 independent reflections
Radiation source: fine-focus sealed tube	3641 reflections with *I* > 2σ(*I*)
graphite	*R*_int_ = 0.051
Detector resolution: 13.6612 pixels mm^-1^	θ_max_ = 27.5°, θ_min_ = 3.2°
CCD profile fitting scans	*h* = −13→13
Absorption correction: multi-scan (*CrystalClear*; Rigaku, 2005)	*k* = −13→13
*T*_min_ = 0.90, *T*_max_ = 0.99	*l* = −14→14
11276 measured reflections	

### Refinement

**Table:**

Refinement on *F*^2^	Primary atom site location: structure-invariant direct methods
Least-squares matrix: full	Secondary atom site location: difference Fourier map
*R*[*F*^2^ > 2σ(*F*^2^)] = 0.055	Hydrogen site location: inferred from neighbouring sites
*wR*(*F*^2^) = 0.157	H-atom parameters constrained
*S* = 1.08	*w* = 1/[σ^2^(*F*_o_^2^) + (0.0745*P*)^2^ + 0.0901*P*] where *P* = (*F*_o_^2^ + 2*F*_c_^2^)/3
4940 reflections	(Δ/σ)_max_ < 0.001
299 parameters	Δρ_max_ = 0.30 e Å^−^^3^
0 restraints	Δρ_min_ = −0.37 e Å^−^^3^

### Special details

**Table:**

Geometry. All esds (except the esd in the dihedral angle between two l.s. planes) are estimated using the full covariance matrix. The cell esds are taken into account individually in the estimation of esds in distances, angles and torsion angles; correlations between esds in cell parameters are only used when they are defined by crystal symmetry. An approximate (isotropic) treatment of cell esds is used for estimating esds involving l.s. planes.
Refinement. Refinement of F^2^ against ALL reflections. The weighted R-factor wR and goodness of fit S are based on F^2^, conventional R-factors R are based on F, with F set to zero for negative F^2^. The threshold expression of F^2^ > 2sigma(F^2^) is used only for calculating R-factors(gt) etc. and is not relevant to the choice of reflections for refinement. R-factors based on F^2^ are statistically about twice as large as those based on F, and R- factors based on ALL data will be even larger.

### Fractional atomic coordinates and isotropic or equivalent isotropic displacement parameters (Å^2^)

**Table:**

	*x*	*y*	*z*	*U*_iso_*/*U*_eq_	
S1	0.29772 (5)	0.18203 (5)	−0.09359 (5)	0.03453 (17)	
O1	0.3379 (2)	0.27308 (18)	−0.20841 (17)	0.0687 (6)	
O2	0.21525 (15)	0.08446 (15)	−0.10027 (16)	0.0436 (4)	
O3	0.41568 (16)	0.11518 (16)	−0.05151 (17)	0.0505 (4)	
C18	0.1835 (2)	0.2733 (2)	0.0305 (2)	0.0327 (5)	
C19	0.1698 (2)	0.4098 (2)	0.0133 (2)	0.0403 (5)	
H19A	0.2168	0.4545	−0.0658	0.039 (6)*	
C20	0.0866 (3)	0.4795 (2)	0.1134 (3)	0.0477 (6)	
H20A	0.0786	0.5712	0.1011	0.049 (7)*	
C21	0.0147 (3)	0.4160 (2)	0.2312 (3)	0.0496 (6)	
C22	0.0275 (3)	0.2787 (3)	0.2467 (3)	0.0536 (6)	
H22A	−0.0212	0.2340	0.3252	0.053 (7)*	
C23	0.1107 (3)	0.2083 (2)	0.1477 (2)	0.0455 (6)	
H23A	0.1182	0.1166	0.1596	0.052 (7)*	
C24	−0.0759 (4)	0.4946 (4)	0.3410 (4)	0.0796 (10)	
H24A	−0.1174	0.4350	0.4155	0.107 (14)*	
H24B	−0.1503	0.5540	0.3170	0.154 (19)*	
H24C	−0.0173	0.5443	0.3597	0.107 (13)*	
O4	0.93542 (15)	0.17176 (15)	−0.06761 (14)	0.0415 (4)	
H4A	1.0199	0.1458	−0.0778	0.062*	
N1	0.61227 (17)	0.16841 (15)	0.05577 (15)	0.0299 (4)	
H1A	0.6871	0.1067	0.0593	0.045*	
H1B	0.5473	0.1276	0.0460	0.045*	
C1	0.9083 (2)	0.1638 (2)	−0.17555 (19)	0.0321 (4)	
C2	1.0121 (2)	0.1192 (2)	−0.2840 (2)	0.0416 (5)	
H2B	1.1059	0.0937	−0.2862	0.048 (7)*	
C3	0.9745 (3)	0.1129 (3)	−0.3888 (2)	0.0510 (6)	
H3B	1.0438	0.0832	−0.4619	0.063 (8)*	
C4	0.8365 (3)	0.1497 (3)	−0.3867 (2)	0.0549 (7)	
H4B	0.8124	0.1436	−0.4575	0.062 (8)*	
C5	0.7330 (2)	0.1963 (2)	−0.2791 (2)	0.0440 (6)	
H5A	0.6394	0.2210	−0.2778	0.043 (6)*	
C6	0.7681 (2)	0.2058 (2)	−0.17350 (19)	0.0326 (5)	
C7	0.6613 (2)	0.2667 (2)	−0.06062 (19)	0.0342 (5)	
H7A	0.5793	0.3126	−0.0840	0.041 (6)*	
H7B	0.7030	0.3318	−0.0398	0.037 (6)*	
C8	0.5474 (2)	0.22312 (18)	0.18374 (18)	0.0291 (4)	
C9	0.6647 (2)	0.2556 (2)	0.2224 (2)	0.0426 (5)	
H9A	0.7140	0.3223	0.1588	0.044 (7)*	
H9B	0.7333	0.1770	0.2278	0.051 (7)*	
C10	0.5976 (3)	0.3071 (3)	0.3537 (2)	0.0566 (7)	
H10A	0.6724	0.3291	0.3790	0.098 (11)*	
C11	0.4734 (3)	0.1157 (2)	0.2835 (2)	0.0458 (6)	
H11A	0.5417	0.0370	0.2889	0.043 (6)*	
H11B	0.3995	0.0927	0.2592	0.054 (7)*	
C12	0.4893 (3)	0.4311 (3)	0.3442 (3)	0.0554 (7)	
H12A	0.4482	0.4651	0.4261	0.076 (9)*	
H12B	0.5367	0.4990	0.2807	0.065 (8)*	
C13	0.3727 (3)	0.3962 (2)	0.3060 (2)	0.0483 (6)	
H13A	0.3030	0.4752	0.3006	0.069 (8)*	
C14	0.4387 (2)	0.3461 (2)	0.1752 (2)	0.0380 (5)	
H14A	0.3649	0.3249	0.1495	0.052 (7)*	
H14B	0.4851	0.4143	0.1114	0.040 (6)*	
C15	0.2985 (3)	0.2895 (3)	0.4054 (2)	0.0572 (7)	
H15A	0.2242	0.2672	0.3811	0.070 (9)*	
H15B	0.2548	0.3214	0.4882	0.062 (8)*	
C16	0.4079 (3)	0.1676 (3)	0.4138 (2)	0.0565 (7)	
H16A	0.3603	0.0989	0.4780	0.073 (9)*	
C17	0.5229 (4)	0.2011 (3)	0.4530 (3)	0.0684 (8)	
H17A	0.5911	0.1227	0.4600	0.085 (10)*	
H17B	0.4812	0.2328	0.5360	0.065 (8)*	

### Atomic displacement parameters (Å^2^)

**Table:**

	*U*^11^	*U*^22^	*U*^33^	*U*^12^	*U*^13^	*U*^23^
S1	0.0308 (3)	0.0307 (3)	0.0430 (3)	−0.0026 (2)	−0.0129 (2)	−0.0076 (2)
O1	0.0865 (14)	0.0502 (11)	0.0455 (10)	−0.0061 (10)	0.0014 (9)	0.0004 (9)
O2	0.0344 (8)	0.0404 (8)	0.0655 (10)	−0.0005 (7)	−0.0227 (7)	−0.0227 (8)
O3	0.0351 (8)	0.0495 (10)	0.0789 (12)	0.0051 (7)	−0.0306 (8)	−0.0252 (9)
C18	0.0311 (10)	0.0294 (10)	0.0428 (12)	−0.0043 (8)	−0.0166 (9)	−0.0085 (9)
C19	0.0397 (12)	0.0277 (11)	0.0539 (14)	−0.0037 (9)	−0.0179 (11)	−0.0024 (10)
C20	0.0486 (14)	0.0294 (12)	0.0693 (17)	0.0021 (10)	−0.0239 (13)	−0.0161 (12)
C21	0.0429 (13)	0.0504 (14)	0.0610 (16)	0.0021 (11)	−0.0185 (12)	−0.0272 (13)
C22	0.0560 (15)	0.0543 (15)	0.0443 (14)	−0.0132 (13)	−0.0046 (12)	−0.0103 (12)
C23	0.0544 (14)	0.0322 (12)	0.0467 (13)	−0.0105 (11)	−0.0103 (11)	−0.0057 (10)
C24	0.071 (2)	0.083 (2)	0.085 (2)	0.000 (2)	−0.0134 (18)	−0.050 (2)
O4	0.0315 (8)	0.0535 (10)	0.0448 (9)	−0.0014 (7)	−0.0160 (7)	−0.0172 (8)
N1	0.0286 (8)	0.0274 (8)	0.0342 (9)	−0.0034 (7)	−0.0091 (7)	−0.0083 (7)
C1	0.0308 (10)	0.0328 (11)	0.0344 (11)	−0.0092 (9)	−0.0093 (8)	−0.0067 (9)
C2	0.0312 (11)	0.0471 (13)	0.0451 (13)	−0.0077 (10)	−0.0072 (10)	−0.0110 (11)
C3	0.0440 (13)	0.0687 (17)	0.0370 (13)	−0.0114 (12)	−0.0017 (11)	−0.0187 (12)
C4	0.0516 (15)	0.085 (2)	0.0323 (12)	−0.0164 (14)	−0.0124 (11)	−0.0139 (13)
C5	0.0361 (12)	0.0604 (15)	0.0356 (12)	−0.0084 (11)	−0.0118 (10)	−0.0058 (11)
C6	0.0301 (10)	0.0341 (11)	0.0328 (10)	−0.0062 (8)	−0.0081 (8)	−0.0050 (9)
C7	0.0315 (11)	0.0318 (11)	0.0368 (11)	−0.0030 (9)	−0.0092 (9)	−0.0042 (9)
C8	0.0304 (10)	0.0266 (10)	0.0315 (10)	−0.0024 (8)	−0.0102 (8)	−0.0087 (8)
C9	0.0385 (12)	0.0485 (14)	0.0477 (13)	−0.0038 (11)	−0.0202 (10)	−0.0135 (11)
C10	0.0604 (16)	0.0713 (18)	0.0536 (15)	−0.0102 (14)	−0.0294 (13)	−0.0232 (14)
C11	0.0560 (15)	0.0351 (12)	0.0390 (12)	−0.0112 (11)	−0.0027 (11)	−0.0087 (10)
C12	0.0723 (18)	0.0459 (14)	0.0525 (16)	−0.0113 (13)	−0.0156 (13)	−0.0245 (13)
C13	0.0493 (14)	0.0387 (13)	0.0528 (14)	0.0085 (11)	−0.0133 (12)	−0.0188 (11)
C14	0.0381 (12)	0.0351 (11)	0.0410 (12)	0.0034 (9)	−0.0155 (10)	−0.0097 (10)
C15	0.0503 (15)	0.0623 (17)	0.0482 (15)	−0.0076 (13)	0.0052 (12)	−0.0250 (13)
C16	0.0745 (18)	0.0446 (14)	0.0359 (13)	−0.0130 (13)	0.0009 (12)	−0.0048 (11)
C17	0.096 (2)	0.0683 (19)	0.0377 (14)	0.0072 (18)	−0.0260 (15)	−0.0122 (14)

### Geometric parameters (Å, °)

**Table:**

S1—O1	1.4319 (18)	C5—C6	1.384 (3)
S1—O3	1.4468 (16)	C5—H5A	0.9300
S1—O2	1.4675 (15)	C6—C7	1.498 (3)
S1—C18	1.764 (2)	C7—H7A	0.9700
C18—C23	1.382 (3)	C7—H7B	0.9700
C18—C19	1.383 (3)	C8—C9	1.520 (3)
C19—C20	1.377 (3)	C8—C14	1.527 (3)
C19—H19A	0.9300	C8—C11	1.528 (3)
C20—C21	1.378 (3)	C9—C10	1.542 (3)
C20—H20A	0.9300	C9—H9A	0.9700
C21—C22	1.392 (4)	C9—H9B	0.9701
C21—C24	1.523 (4)	C10—C17	1.518 (4)
C22—C23	1.374 (3)	C10—C12	1.534 (4)
C22—H22A	0.9300	C10—H10A	0.9799
C23—H23A	0.9300	C11—C16	1.533 (3)
C24—H24A	0.9600	C11—H11A	0.9700
C24—H24B	0.9601	C11—H11B	0.9699
C24—H24C	0.9599	C12—C13	1.523 (4)
O4—C1	1.360 (2)	C12—H12A	0.9700
O4—H4A	0.8200	C12—H12B	0.9700
N1—C7	1.497 (2)	C13—C15	1.521 (4)
N1—C8	1.524 (2)	C13—C14	1.530 (3)
N1—H1A	0.9000	C13—H13A	0.9800
N1—H1B	0.8999	C14—H14A	0.9700
C1—C2	1.386 (3)	C14—H14B	0.9700
C1—C6	1.397 (3)	C15—C16	1.524 (4)
C2—C3	1.381 (3)	C15—H15A	0.9699
C2—H2B	0.9300	C15—H15B	0.9700
C3—C4	1.370 (4)	C16—C17	1.508 (4)
C3—H3B	0.9301	C16—H16A	0.9799
C4—C5	1.385 (3)	C17—H17A	0.9700
C4—H4B	0.9300	C17—H17B	0.9700
			
O1—S1—O3	113.98 (12)	C9—C8—N1	109.63 (16)
O1—S1—O2	113.46 (11)	C9—C8—C14	110.36 (17)
O3—S1—O2	109.68 (10)	N1—C8—C14	110.60 (16)
O1—S1—C18	107.64 (11)	C9—C8—C11	109.08 (18)
O3—S1—C18	105.85 (10)	N1—C8—C11	107.61 (15)
O2—S1—C18	105.56 (9)	C14—C8—C11	109.50 (18)
C23—C18—C19	119.4 (2)	C8—C9—C10	108.90 (18)
C23—C18—S1	119.99 (16)	C8—C9—H9A	109.9
C19—C18—S1	120.54 (17)	C10—C9—H9A	109.9
C20—C19—C18	119.9 (2)	C8—C9—H9B	109.9
C20—C19—H19A	120.1	C10—C9—H9B	109.9
C18—C19—H19A	120.0	H9A—C9—H9B	108.3
C19—C20—C21	121.4 (2)	C17—C10—C12	109.5 (2)
C19—C20—H20A	119.3	C17—C10—C9	109.4 (2)
C21—C20—H20A	119.3	C12—C10—C9	109.5 (2)
C20—C21—C22	118.1 (2)	C17—C10—H10A	109.5
C20—C21—C24	120.6 (2)	C12—C10—H10A	109.5
C22—C21—C24	121.2 (3)	C9—C10—H10A	109.5
C23—C22—C21	120.9 (2)	C8—C11—C16	108.81 (18)
C23—C22—H22A	119.5	C8—C11—H11A	109.9
C21—C22—H22A	119.5	C16—C11—H11A	109.9
C22—C23—C18	120.2 (2)	C8—C11—H11B	110.0
C22—C23—H23A	119.9	C16—C11—H11B	109.9
C18—C23—H23A	119.9	H11A—C11—H11B	108.3
C21—C24—H24A	109.4	C13—C12—C10	109.3 (2)
C21—C24—H24B	109.5	C13—C12—H12A	109.9
H24A—C24—H24B	109.5	C10—C12—H12A	109.9
C21—C24—H24C	109.5	C13—C12—H12B	109.8
H24A—C24—H24C	109.5	C10—C12—H12B	109.7
H24B—C24—H24C	109.5	H12A—C12—H12B	108.3
C1—O4—H4A	109.4	C15—C13—C12	109.8 (2)
C7—N1—C8	116.28 (14)	C15—C13—C14	109.65 (19)
C7—N1—H1A	108.2	C12—C13—C14	109.3 (2)
C8—N1—H1A	108.2	C15—C13—H13A	109.3
C7—N1—H1B	108.2	C12—C13—H13A	109.3
C8—N1—H1B	108.2	C14—C13—H13A	109.4
H1A—N1—H1B	107.4	C8—C14—C13	109.29 (17)
O4—C1—C2	123.22 (19)	C8—C14—H14A	109.9
O4—C1—C6	116.32 (18)	C13—C14—H14A	109.8
C2—C1—C6	120.45 (19)	C8—C14—H14B	109.8
C3—C2—C1	119.2 (2)	C13—C14—H14B	109.8
C3—C2—H2B	120.3	H14A—C14—H14B	108.3
C1—C2—H2B	120.4	C13—C15—C16	108.9 (2)
C4—C3—C2	120.9 (2)	C13—C15—H15A	109.9
C4—C3—H3B	119.5	C16—C15—H15A	109.8
C2—C3—H3B	119.5	C13—C15—H15B	110.0
C3—C4—C5	119.9 (2)	C16—C15—H15B	109.9
C3—C4—H4B	120.0	H15A—C15—H15B	108.3
C5—C4—H4B	120.1	C17—C16—C15	110.2 (2)
C6—C5—C4	120.4 (2)	C17—C16—C11	110.0 (2)
C6—C5—H5A	119.8	C15—C16—C11	109.6 (2)
C4—C5—H5A	119.8	C17—C16—H16A	109.0
C5—C6—C1	119.01 (19)	C15—C16—H16A	109.0
C5—C6—C7	121.43 (19)	C11—C16—H16A	109.0
C1—C6—C7	119.47 (18)	C16—C17—C10	109.3 (2)
N1—C7—C6	113.46 (16)	C16—C17—H17A	109.8
N1—C7—H7A	108.9	C10—C17—H17A	109.9
C6—C7—H7A	108.9	C16—C17—H17B	109.7
N1—C7—H7B	108.9	C10—C17—H17B	109.8
C6—C7—H7B	108.9	H17A—C17—H17B	108.3
H7A—C7—H7B	107.7		

### Hydrogen-bond geometry (Å, °)

**Table:**

Cg is the centroid of the C18–C23 benzene ring.

**Table:**

*D*—H···*A*	*D*—H	H···*A*	*D*···*A*	*D*—H···*A*
N1—H1A···O2^i^	0.90	2.09	2.922 (2)	154.
N1—H1B···O3	0.90	2.05	2.860 (2)	150.
O4—H4A···O2^ii^	0.82	1.90	2.719 (2)	180.
C14—H14A···Cg	0.97	2.75	3.668 (3)	158

Symmetry codes: (i) −*x*+1, −*y*, −*z*; (ii) *x*+1, *y*, *z*.
